# Profile of the Change in Depression during Proton-Pump Inhibitor Therapy in Patients with Gastroesophageal Reflux Disease: Influence of the Mucosal Break

**DOI:** 10.3390/ijerph18115964

**Published:** 2021-06-02

**Authors:** Chia-Liang Wu, Chien-Lin Chen, Shu-Hui Wen

**Affiliations:** 1 Department of Psychiatry, Taipei Veterans General Hospital, Yuli Branch, Hualien 981002, Taiwan; peterwu2000.tw@yahoo.com.tw; 2 Institute of Medical Sciences, Tzu Chi University, Hualien 970374, Taiwan; 3 Department of Internal Medicine, Buddhist Tzu Chi General Hospital, Hualien 970473, Taiwan; harry.clchen@msa.hinet.net; 4 Department of Public Health, College of Medicine, Tzu Chi University, Hualien 970374, Taiwan

**Keywords:** gastroesophageal reflux disease, depression, proton-pump inhibitors, prospective cohort study, odds ratio

## Abstract

Given the frequent concomitance between depression and gastroesophageal reflux disease (GERD), it is important to evaluate the change of depression in patients with GERD, especially considering the presence of esophageal mucosal breaks (MB). This study aimed to examine the change in the levels of depression in patients with GERD during proton-pump inhibitors (PPI) therapy. We designed a prospective cohort study to explore the profile of the alteration in depression with respect to the impact of esophageal MB. This study recruited 172 eligible patients with GERD between February 2016 and May 2018. The change in depression was defined as the difference between the respective Taiwanese Depression Questionnaire (TDQ) scores obtained at baseline and after PPI therapy. Multivariate linear regression models were used to estimate the factors associated with the change in depression. The results revealed statistically significant improvements in the TDQ score (mean score: baseline = 13.2, after PPI therapy = 10.9, *p* < 0.01, Cohen’s d = 0.30) during PPI therapy for GERD. Moreover, the MB was an independent variable associated with changes in the TDQ score [B = 3.31, 95% confidence interval (CI): (1.12, 5.51), *p* < 0.01] and the improvement in depression [odds ratio = 0.38, 95% CI: (0.17, 0.86), *p* = 0.02]. Our findings revealed that depressive symptoms improved slightly following PPI therapy. Moreover, MB was an unfavorable prognostic factor for the improvement in depression.

## 1. Introduction

Gastroesophageal reflux disease (GERD) is a digestive disorder characterized by the flow of the acidic content of the stomach back into the esophagus. The typical symptoms of GERD include heartburn, acid regurgitation, and an acidic taste in the mouth. The prevalence of GERD is estimated to be 10–20% in Western countries [1] and 2.5–7.8% in East Asia [2,3]. The health ramifications of GERD include asthma [4], chronic cough [5], esophageal cancer [6], and depression [7], deterioration in the quality of life [3,8], and decrease work productivity [9]. Proton-pump inhibitors (PPI), which inhibit the hydrogen/potassium adenosine triphosphatase signaling pathway in the gastric parietal cells, are the most effective and widely used pharmacotherapy for GERD [10]. GERD can be divided into erosive reflux disease (ERD) and non-erosive reflux disease (NERD) based on the presence of the esophageal mucosal break (MB). NERD accounts for approximately 70% of cases of GERD [11], and its pathophysiology involves not only peripheral factors (mucosal, luminal, etc.) but also central factors (psychological stress, etc.) [12]. Previous cross-sectional studies found that NERD was associated with depression [13,14,15]. Thus, the influence of MB on depression in patients with GERD patients deserves further investigation.

Previous studies revealed that the frequency of comorbidity of depressive disorders in patients with GERD is approximately 30–65% [16]. The severity of depression was significantly higher in GERD participants than in the controls [15]. A nested case-control study also demonstrated bidirectional associations between GERD and depression [17]. These associations between GERD and depression have necessitated investigations into whether PPI therapy can contribute to the improvement in depression in patients with GERD.

Numerous previous studies investigated the relationships between GERD and depression [7,13,15,17,18,19,20,21]. Moreover, cross-sectional [22] and retrospective [23] studies stated that PPI might be associated with an increased risk of developing depression. However, studies focusing on the change in depressive symptoms before and after PPI therapy are absent. Evaluating the depression status during PPI therapy in clinical settings can contribute to developing a comprehensive therapeutic strategy for depressive symptoms. Hence, we designed and conducted a prospective cohort study to examine the change in the depressive and reflux symptoms before and after PPI therapy in patients with GERD. This study also explored the relationship between MB and the change in depression during PPI therapy.

## 2. Materials and Methods

### 2.1. Study Design

**Figure 1:**
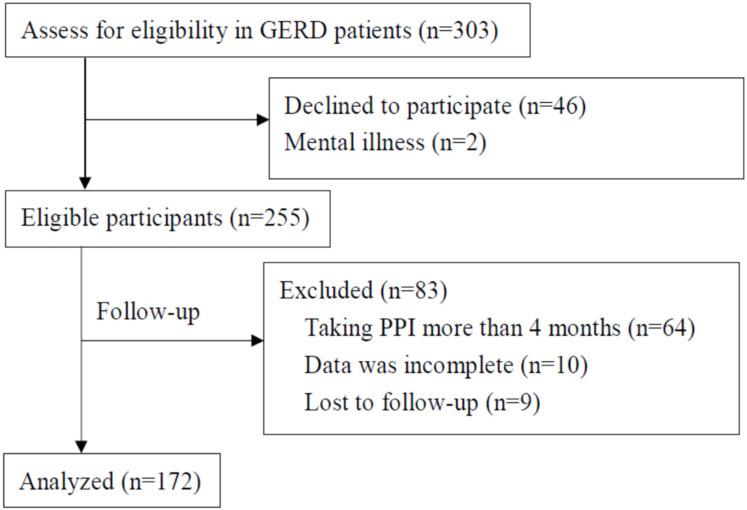
Flowchart depicting sample selection for the study. GERD, gastroesophageal reflux disease; PPI, proton-pump inhibitors.

We conducted a prospective cohort study of patients with GERD who underwent upper gastrointestinal (UGI) endoscopy at the gastroenterology clinics of Hualien Tzu Chi Hospital between February 2016 and May 2018. Patients with GERD were defined as those who complained of symptoms such as acid regurgitation and heartburn, which was identified using the reflux disease questionnaire (RDQ) score ≥ 3 [24] (this procedure is described in greater detail in the subsequent subsection). Initially, 303 GERD participants aged above 20 years who had not taken PPIs within 1 year before enrollment were recruited. Exclusion criteria included: (1) mild, severe, or uncontrolled mental illness, (2) malignant tumor, and (3) pregnancy. Forty-eight of these 303 patients declined to participate (n = 46) or had mild, severe, or uncontrolled mental illness (n = 2), which rendered them ineligible for enrollment. A total of 255 participants started PPI therapy and were followed up for a maximum of 4 months, owing to the payment regulations of the Taiwanese national insurance agency for PPI therapy. The start date and end of follow-up were the dates of the first and last prescription of PPIs, respectively. During follow-up, 83 participants were excluded because they took PPIs for more than 4 months due to severe GERD (n = 64), their data was incomplete (n = 10), or were lost to follow-up (n = 9). Finally, 172 participants were included in this prospective cohort study after a follow-up of up to 4 months. Figure 1 depicts the flowchart of the selection process of this prospective study, which was approved by the Ethics Committee of Hualien Tzu Chi Hospital, Buddhist Tzu Chi Medical Foundation (IRB105-145-B). Each participant provided written informed consent.

### 2.2. Measurements

At baseline, participants were asked to complete questionnaires related to sociodemographic variables, including age, sex, marriage (yes or no), level of education (<university or ≥university), body mass index (BMI), alcohol consumption (yes: at least once per month; no: 0 per month), coffee intake (yes: at least four days per week; no: at most three days per week), tea intake (yes: at least four days per week; no: at most three days per week), and exercise (yes or no). The presence or absence of an esophageal MB was identified according to the results of the UGI endoscopy. All study participants completed questionnaires, including the RDQ, Taiwanese Depression Questionnaire (TDQ), and Pittsburgh Sleep Quality Index (PSQI) at baseline. The duration of PPI therapy was calculated as the sum of all PPI prescription days during the follow-up period. Participants were asked to complete the RDQ and TDQ again after PPI therapy.

The RDQ was designed to examine the frequency and severity of symptoms, such as heartburn, acid regurgitation, and dyspepsia. The Mandarin version of this self-administered questionnaire exhibited credible reliability and constructed validity [25]. The RDQ includes 12 questions that aim to measure the frequency, severity, and duration of the following symptoms: (1) acidic taste in the mouth and upward movement of the content of the stomach (regurgitation scale), (2) pain or burning in the region behind the breastbone (heartburn scale), and (3) pain or burning sensation in the upper stomach region (dyspepsia scale) [26]. Response options are scaled as Likert-type with scores ranging from 0 to 5 for frequency (not present to daily) and severity (not present to severe). The heartburn and regurgitation subscales can be combined into a GERD dimension [26,27]. In this study, the RDQ score was calculated as the sum of eight non-dyspepsia-related questions from the RDQ questionnaire. The RDQ score ≥ 3 is defined as having troublesome reflux symptoms [24]. A higher RDQ score represents a higher level of reflux symptoms. The change in reflux symptoms (∆RDQ = RDQ_t1_−RDQ_t0_) was defined as the difference between the respective RDQ scores measured at baseline (RDQ_t0_) and after PPI therapy (RDQ_t1_). A negative ∆RDQ score indicated the improvement in reflux after PPI therapy and vice versa.

The TDQ is a culture-sensitive instrument that can be used to measure depression in Taiwan. It has a Cronbach’s α of 0.90, satisfactory concurrent validity, a sensitivity of 0.89, and specificity of 0.92 at a cutoff score of 19 [28]. It comprises 18 self-reported items related to mood, sleeping problems, appetite, energy, interest in regular activities, crying, feelings about the future, etc. Participants were asked to rate each item on a scale from 0 to 3 based on the following question: “How often did you feel physical and emotional anguish during the past week?”. The TDQ score ranges from 0 to 54, with a higher score indicating greater severity of depression.

The PSQI is a self-rated questionnaire that assesses sleep quality and disturbances over a one-month interval. Nineteen individual items are used to generate seven “component” scores: subjective sleep quality, sleep latency, sleep duration, habitual sleep efficiency, sleep disturbances, use of sleeping medication, and daytime dysfunction. Scores for each component range from 0 to 3. The sum of the scores of these seven components yields one global score (range: 0 to 21). A higher score indicates greater sleep disturbance, and the score ≥ 5 indicates poor sleep quality [29].

### 2.3. Study Outcomes

The primary outcome of this study was the change in depression measured by the change in the TDQ score (∆TDQ hereafter). The TDQ scores were measured at baseline (TDQ_t0_) and obtained at the end of follow-up (TDQ_t1_), i.e., ∆TDQ = TDQ_t1_−TDQ_t0_. A negative ∆TDQ score indicated the improvement in the depressive symptoms after PPI therapy and vice versa. The binary response variable was the secondary outcome, coded as 1 or 0 for patients with or without improvement in the depressive symptoms, i.e., 1: ∆TDQ < 0; 0: ∆TDQ ≥ 0, respectively.

### 2.4. Statistical Analysis

Descriptive analyses were performed to demonstrate the characteristics of the participants. Kolmogorov-Smirnov test was used to check the normality of continuous data. Categorical data were presented as numbers and percentages, whereas continuous data were presented as the median and interquartile range (IQR). The Chi-squared or Mann–Whitney U test was used to compare participants’ categorical or continuous variables with MB (termed the MB group) and without MB (termed the NoMB group). The Wilcoxon signed-rank test was used to compare the ∆TDQ and ∆RDQ before and after PPI therapy in patients with GERD. Cohen’s d was applied to quantify the magnitude for the change of the scores. The value of Cohen’s d was considered as small (0.2 to 0.5), intermediate (0.5 to 0.8) and large (>0.8) [30].

To identify the factors associated with the change in the TDQ scores, multivariate linear regression models, which were adjusted for baseline characteristics such as MB, sex, age, level of education, marriage, BMI, alcohol consumption, coffee intake, tea intake, exercise, PPI duration, and the RDQ_t0_ and PSQI scores, were used. Moreover, we performed residual analysis to check the validity of the assumptions of a linear regression model. Five outliers (the absolute value of standardized residuals is greater than three) were detected in the residual analysis. After removing these outliers, the residuals analysis revealed that normality and equal variance were met. We used the dataset after removing 5 outliers to construct the final multivariate linear regression model.

Additionally, multivariate logistic regression models were used to identify the factors associated with the secondary outcome variable, improvement in depressive symptoms after PPI therapy. The Hosmer-Lemeshow test showed that the goodness of fit for this model was adequate. *p*-values ˂ 0.05 were considered statistically significant. All analyses were conducted using SPSS (version 19; IBM Corp., Armonk, NY, USA) software.

## 3. Results

### 3.1. Sample Characteristics

We conducted a prospective cohort study with data collected from 172 participants with a mean follow-up of 51.4 days. These participants were classified into the MB (n = 52, 30.2%) and NoMB groups (n = 120, 69.8%). During follow-up, the duration of PPI therapy was longer in the MB group than in the NoMB group (median ± IQR = 60.0 ± 54.0 vs. 40.0 ± 41.0 days, respectively; *p* < 0.01). The MB group had a significantly lower proportion of women (44.2% vs. 69.2%, *p* < 0.01), and a higher rate of alcohol consumption (53.8% vs. 34.2%, *p* = 0.02) compared to the NoMB group. However, most baseline characteristics were similar between the MB and NoMB groups, as shown in Table 1. Moreover, significant differences were not observed in the baseline symptom scores of the RDQ_t0_ (12.0 ± 15.0 vs. 12.5 ± 13.0, *p* = 0.66), TDQ_t0_ (10.0 ± 14.0 vs. 11.0 ± 12.0, *p* = 0.07), and PSQI (6.0 ± 4.0 vs. 5.0 ± 5.0, *p* = 0.95).

### 3.2. Change in Reflux Symptoms and Depression during Follow-Up

The overall average RDQ score decreased after PPI therapy (14.8 to 1.9; ∆RDQ: −12.9, *p* < 0.01, Cohen’s d: 1.12). Significant improvements were observed in the reflux symptoms after PPI therapy in the two groups compared to baseline (MB group: ∆RDQ = −13.2, *p* < 0.01, Cohen’s d: 1.24; NoMB group: ∆RDQ = −12.7, *p* < 0.01, Cohen’s d: 1.08). In other words, the RDQ scores decreased after PPI therapy in more than 95% of patients in both groups (MB group: 96.2%; NoMB group: 95.0%, *p* = 0.99). The ∆RDQ score did not differ between the MB and NoMB groups (∆RDQ score: −13.2 and −12.7 in the MB and NoMB groups, respectively; *p* = 0.66) (Table 2).

**Figure 2:**
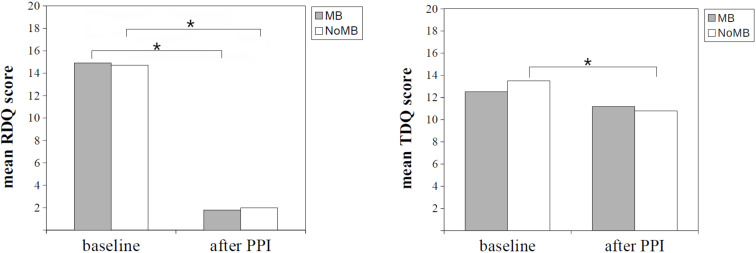
Bar chart illustrating the RDQ and TDQ scores before and after PPI therapy. MB, mucosal break; NoMB, no mucosal break; PPI, proton-pump inhibitors; RDQ, reflux disease questionnaire; TDQ, Taiwanese depression questionnaire. * *p*-value < 0.05.

**Figure 3:**
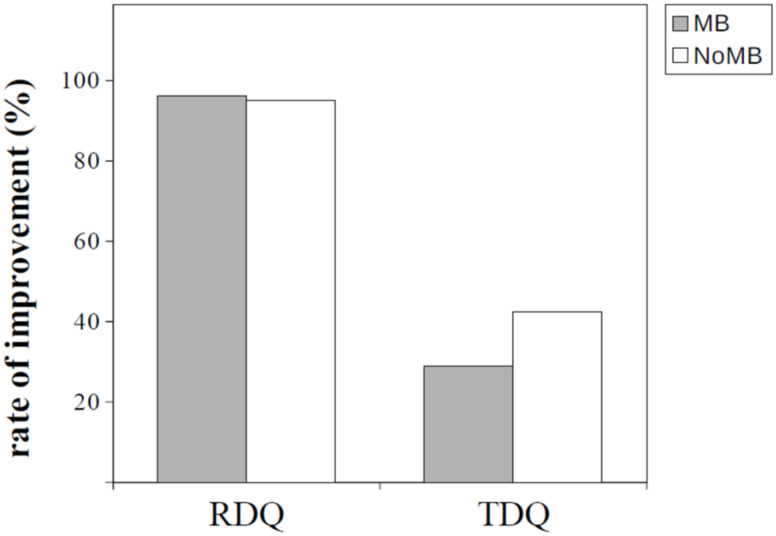
Bar chart illustrating the rate of improvement in the RDQ and TDQ scores in the MB and NoMB groups. MB, mucosal break; NoMB, no mucosal break; RDQ, reflux disease questionnaire; TDQ, Taiwanese depression questionnaire.

The TDQ scores (used to assess the depressive symptoms) decreased significantly in the whole sample (13.2 to 10.9, *p* < 0.01, Cohen’s d: 0.30), as well as the NoMB group (13.5 to 10.8, *p* < 0.01 Cohen’s d: 0.38) during the follow-up period. However, the change in depression was not significant in the MB group (12.5 to 11.2, *p* = 0.28, Cohen’s d: 0.15). Moreover, the rate of improvement in ∆TDQ score was 28.8% in the MB group and 42.5% in the NoMB group (*p* = 0.12). The ∆TDQ scores reached a marginally significant difference between the MB and NoMB groups (∆TDQ score: −1.2 and −2.7 in the MB and NoMB groups, respectively; *p* = 0.07) (Table 2). Figure 2 and Figure 3 illustrate the results mentioned above in the form of bar charts.

### 3.3. Factors Associated with the Change in Depression

The change in depression, as evidenced by the ∆TDQ, was the primary outcome of this study. Table 3 presents the factors associated with the change in depression elicited using the univariate and multivariate linear regression models. Univariate analysis revealed that education above the university level [B = −3.01, 95% confidence interval (CI): (−5.23, −0.80), *p* = 0.01], baseline RDQ score [B = −0.14, 95% CI: (−0.27, −0.01), *p* = 0.03], and sleep quality PSQI score [B = −0.41, 95% CI: (−0.73, −0.09), *p* = 0.01) were associated with the change in depression. After adjusting for other factors, multivariate analysis revealed that MB [B = 3.31, 95% CI: (1.12, 5.51), *p* < 0.01] and being female [B = 3.74, 95% CI: (1.59, 5.89), *p* < 0.01] were unfavorable factors for the change in depression. Education above university level [B = −3.08, 95% CI: (−5.10, −1.06), *p* < 0.01], longer duration of PPI therapy [B = −0.04, 95% CI: (−0.07, −0.01), *p* = 0.02], and the PSQI score [B = −0.61, 95% CI: (−0.90, −0.31), *p* < 0.01] were independent favorable factors for the change in depression.

The secondary outcome was the binary variable indicating the improvement in depression. Table 4 presents the results of univariate and multivariate logistic regression models, which revealed the factors associated with the improvement in depression. Univariate analysis revealed that only the duration of PPI therapy [odds ratio (OR) = 1.01, 95% CI: (1.00, 1.02), *p* = 0.04) was associated with the improvement in depression. Multivariate analysis revealed that MB was an independent unfavorable factor for the improvement in depression (OR = 0.38, 95% CI: (0.17, 0.86), *p* = 0.02] after adjusting for other factors. However, education above university level (OR = 2.20, 95% CI: (1.07, 4.51), *p* = 0.03), longer duration of PPI therapy [OR = 1.01, 95% CI: (1.00, 1.03), *p* = 0.01], and higher PSQI score [OR = 1.11, 95% CI: (1.00, 1.24), *p* = 0.04] were independent favorable factors for the improvement in depression.

## 4. Discussion

This was the first study to examine the change in depression in patients with GERD during PPI therapy. This prospective cohort study found that reflux symptoms improved after PPI therapy, as expected, irrespective of the presence of esophageal MB. However, the depressive symptoms improved only in GERD patients without MB. The rate of improvement of depression was higher in the NoMB group than that in the MB group (42.5% vs. 28.8%, *p* = 0.12). Moreover, esophageal MB was an independent unfavorable factor for the change in depression and the improvement in depression.

A previous nested case-control study found a bidirectional relationship between GERD and depression [17]. However, whether the improvement in GERD symptoms under PPI therapy can improve depression remains unknown. We inferred that the improvement in depression was slight during PPI therapy based on the value of ∆TDQ [mean score: −2.3 in patients with GERD, Cohen’s d: 0.30 (small effect size)] obtained in this study. Meanwhile, the improvement in reflux symptoms [mean ∆RDQ score: −12.9 in patients with GERD, Cohen’s d: 1.12 (large effect size)] was remarkably significant. These findings can possibly be attributed to two factors. First, the longest follow-up time in this study was four months, which was insufficient to evoke a psychological response to the improvement in GERD symptoms. The mechanisms involved in the pathogenesis of GERD are related to various cytokine-related chronic inflammatory processes [31,32]. This process also plays a crucial role in the pathophysiology of mental disorders [33,34]. Thus, the improvement in GERD symptoms may not retard this chronic inflammatory process within a relatively short period. Second, PPI use may be an unfavorable factor for the improvement in depression. Two earlier studies revealed that PPI might be associated with an elevated risk of depression [22,23], probably because PPI-related brain-gut axis dysregulation may contribute to the development of depressive symptoms [35]. Moreover, PPI might decrease the clearance of beta-amyloid in the brain [36], which is associated with depression in older individuals [37].

MB was an unfavorable independent factor for the improvement in depression [adjusted OR = 0.38, 95% CI: (0.17, 0.86), *p* = 0.02] and the change in depression [B = 3.31, 95% CI: (1.12, 5.51), *p* < 0.01]. We could compare these results to those of very few studies due to the lack of clinical studies conducted on this subject. Clinically, esophageal MB is a crucial pathological finding for ERD. In contrast, the diagnosis of NERD entails the absence of visible MB and abnormal esophageal acid exposure measured using the 24-h esophageal pH-monitoring test. Some previous cross-sectional studies [13,14,15] indicated that anxiety and depression might play an important factor in GERD, especially in NERD. It is noteworthy that NERD and ERD appear to have different pathophysiological and clinical characteristics [12]. The psychoneuroimmune interaction, which is associated with emotional function and stress, is considered an important pathophysiological mechanism for NERD [38]. The detailed mechanisms underlying the association between NERD and emotional function and its physiological, anatomical, and histological characteristics remain unclear. Future investigations are necessary to address this issue.

It is worth noting that low sleep quality at baseline, high educational level, and longer duration of PPI prescription were favorable prognostic factors for improvement in depressive symptoms. There has been evidence that sleep disturbance is positively associated with GERD and depression [13,39]. One possible explanation could be patients with GERD symptom relief might improve sleep quality after PPI therapy [40]. Consequently, better sleep quality would save more working hours [41]; thereby leading to improvement of the depression score. Depressive symptoms became better in patients with high educational levels due to mechanisms, including life stress exposure and economic resources [42]. This is in line with previous studies that indicated that low education level is associated with high depressive symptom burden trajectories [43,44]. We also found that a longer duration of PPI therapy significantly improved depression score; however, the magnitude of improvement is slight (e.g., OR = 1.01, 95% CI: (1.00, 1.03) in Table 4). Hence, further studies might be needed to clarify the beneficial effect of a longer PPI prescription period for improving the depression score.

We propose some suggestions for managing the depressive symptoms in GERD patients during PPI therapy based on the results of this study. The rate of the concomitance of depression (TDQ > 19) was 21.5% (37/172) in this study; thus, clinicians should be aware of the mood status of patients with GERD. If the depressive disorder is suspected, clinicians should consider treating it as soon as possible. The TDQ score improved by a value of only 2.3 during PPI therapy if the depressive symptoms were not treated with dedicated anti-depression modalities. Therefore, early intervention strategies, including antidepressants, psychotherapy, and lifestyle modification, should be implemented for depressive disorder. Moreover, the improvement in depression may increase the treatment response of the GERD symptoms to PPIs since depression is one of the factors associated with poor therapeutic response to PPIs [23].

The drug dispensing regulations of the national health insurance program in Taiwan state that PPI can be used for Los Angeles grade A or B in GERD patients for a maximum of 4 months; thus, we only enrolled participants who took PPI for less than four months. For participants who took PPIs for more than four months, it may result from severe GERD or comorbid with other psychological diseases [45]. Hence, we excluded these participants. Inevitably, it impacts the generalizability of our results in real-world clinical settings. Besides, respondents may be too embarrassed to divulge private details or actual symptoms since the sociodemographic, lifestyle, and TDQ, RDQ, and PSQI were self-reported in this study. Although these questionnaires were validated and the participants consented to enroll in this study voluntarily, response bias should be considered. On the other hand, the use of observer-rated depression scales, such as the Hamilton Depression Scales, could have increased the possibility of observer bias [46].

Our study benefitted from the prospective cohort design and validated questionnaires to measure the level of reflux and depressive symptoms. Moreover, all participants underwent UGI endoscopic examinations to identify (presence or absence) of esophageal MB. However, this study had several limitations. First, a substantially longer follow-up duration is desirable to determine the long-term depressive mood status beyond the period of PPI therapy. Second, this study recruited 172 participants. Larger sample size could have conferred greater statistical power for the detection of a more meaningful difference. Third, information on other potential influencing factors (e.g., stress, personality, mood disorders, receiving psychotropic medications, receiving psychotherapy, and social support) that contribute to depression was unavailable in this study.

## 5. Conclusions

In conclusion, this prospective cohort study demonstrated that the level of depression showed mild improvement during PPI therapy in patients with GERD. Moreover, the presence of esophageal MB was an independent unfavorable prognostic factor for depression. Based on the frequent concomitance between depression and GERD, it necessitates evaluating the level of depression with caution in patients with GERD, especially for the presence of esophageal MB.

## Figures and Tables

**Table 1:** Comparison between the characteristics of the MB and NoMB groups.

Baseline Characteristics	Total	MB	NoMB	*p* ^a^
Number	172	52	120	
Female	106 (61.6)	23 (44.2)	83 (69.2)	<0.01
Age (years)	47.5 ± 21.0	44.0 ± 22.0	49.0 ± 22.0	0.20
Level of education				
<University	84 (48.8)	26 (50.0)	58 (48.3)	0.87
≥University	88 (51.2)	26 (50.0)	62 (51.7)	
Marriage	129 (75.0)	37 (71.2)	92 (76.7)	0.45
Body mass index (kg/m^2^)	23.6 ± 5.4	24.3 ± 4.9	23.4 ± 5.0	0.21
Alcohol	69 (40.1)	28 (53.8)	41 (34.2)	0.02
Coffee	78 (45.3)	23 (44.2)	55 (45.8)	0.87
Tea	71 (41.3)	23 (44.2)	48 (40.0)	0.62
Exercise	86 (50.0)	27 (51.9)	59 (49.2)	0.87
RDQ_t0_	12.0 ± 14.0	12.0 ± 15.0	12.5 ± 13.0	0.66
TDQ_t0_	11.0 ± 12.0	10.0 ± 14.0	11.0 ± 12.0	0.07
PSQI	5.0 ± 5.0	6.0 ± 4.0	5.0 ± 5.0	0.95

MB, mucosal break; NoMB, no mucosal break; PSQI, Pittsburgh sleep quality index; RDQ, reflux disease questionnaire; TDQ, Taiwanese depression questionnaire; t_0_, baseline. Categorical data were presented as numbers (percentages), whereas continuous data were presented as the median ± interquartile range. ^a^ Chi-squared or Mann–Whitney U test was used to compare the categorical or continuous variables between MB and NoMB group.

**Table 2:** Change in the TDQ and RDQ scores of patients with GERD before and after PPI therapy.

Measurement	Group	Number			Score (Mean ± Standard Deviation)					
		Total	Improve (%)	*p* ^a^	Baseline (t_0_)	After PPI (t_1_)	Change from Baseline	*p* ^b^	Effect Size (Cohen’s d)	*p* ^c^
RDQ	All	172	164 (95.3)		14.8 ± 8.6	1.9 ± 3.7	−12.9 ± 8.9	<0.01	1.12	
	MB	52	50 (96.2)	0.99	14.9 ± 8.9	1.8 ± 3.5	−13.2 ± 8.6	<0.01	1.24	0.66
	NoMB	120	114 (95.0)		14.7 ± 8.5	2.0 ± 3.7	−12.7 ± 9.1	<0.01	1.08	
TDQ	All	172	66 (38.4)		13.2 ± 10.1	10.9 ± 9.8	−2.3 ± 7.5	<0.01	0.30	
	MB	52	15 (28.8)	0.12	12.5 ± 10.5	11.2 ± 9.2	−1.2 ± 8.1	0.31	0.15	0.07
	NoMB	120	51 (42.5)		13.5 ± 9.9	10.8 ± 10.0	−2.7 ± 7.2	<0.01	0.38	

GERD, gastroesophageal reflux disease; MB, mucosal break; NoMB, no mucosal break; PPI, proton-pump inhibitors; RDQ, reflux disease questionnaire; TDQ, Taiwanese depression questionnaire. ^a^ Chi-squared test was used to compare the rate of improvement in reflux and depressive symptoms between the MB and NoMB groups. ^b^ The Wilcoxon signed-rank was used to compare the score before and after PPI therapy. ^c^ The Mann–Whitney U test was used to compare ∆RDQ and ∆TDQ between the MB and NoMB groups.

**Table 3:** Multivariate linear regression models to examine the factors associated with the change in the Taiwanese Depression Questionnaire score.

Factors	Univariate Model		Multivariate Model ^a^	
	B (n = 172) (95% CI)	*p*	B (n = 167) (95% CI)	*p*
Mucosal break	1.47 (−0.98, 3.92)	0.24	3.31 (1.12, 5.51)	<0.01
Female	2.29 (−0.01, 4.59)	0.05	3.74 (1.59, 5.89)	<0.01
Age (years)	0.06 (−0.02, 0.15)	0.14	0.07 (−0.04, 0.17)	0.21
Education (ref: <university)				
≥University	−3.01 (−5.23, −0.80)	0.01	−3.08 (−5.10, −1.06)	<0.01
Marriage	1.92 (−0.67, 4.52)	0.15	−0.61 (−3.41, 2.19)	0.67
Body mass index (kg/m^2^)	−0.20 (−0.50, 0.10)	0.18	−0.18 (−0.43, 0.08)	0.18
Alcohol	0.67 (−1.64, 2.98)	0.57	1.94 (−0.15, 4.02)	0.07
Coffee	1.55 (−0.71, 3.81)	0.18	0.52 (−1.50, 2.53)	0.56
Tea	−0.88 (−3.18, 1.41)	0.45	−0.18 (−2.18, 1.83)	0.86
Exercise	0.51 (−1.75, 2.77)	0.66	0.24 (−1.72, 2.20)	0.81
PPI duration (days)	−0.02 (−0.06, 0.01)	0.17	−0.04 (−0.07, −0.01)	0.02
RDQ_t0_	−0.14 (−0.27, −0.01)	0.03	−0.07 (−0.19, 0.04)	0.20
PSQI	−0.41 (−0.73, −0.09)	0.01	−0.61 (−0.90, −0.31)	<0.01

CI, confidence interval; PPI, proton pump inhibitors; PSQI, Pittsburgh sleep quality index; RDQ, reflux disease questionnaire. ^a^ Adjusted for the mucosal break, sex, age, level of education, marriage, body mass index, alcohol, coffee, tea, exercise, PPI duration, RDQ_t0_ score, and PSQI score (n = 167 with removing 5 outliers).

**Table 4:** Multivariate logistic regression models to examine the factors associated with the improvement in the Taiwanese Depression Questionnaire score.

Factors	Univariate Model		Multivariate Model ^a^	
	Odds Ratio (95% CI)	*p*	Adjusted Odds Ratio (95% CI)	*p*
Mucosal break	0.55 (0.27, 1.10)	0.09	0.38 (0.17, 0.86)	0.02
Female	0.76 (0.40, 1.42)	0.39	0.58 (0.27, 1.23)	0.15
Age (years)	1.00 (0.97, 1.02)	0.53	0.98 (0.95, 1.01)	0.22
Education (ref: <university)				
≥University	1.86 (1.00, 3.47)	0.05	2.20 (1.07, 4.51)	0.03
Marriage	1.07 (0.52, 2.18)	0.10	2.03 (0.73, 5.66)	0.18
Body mass index (kg/m^2^)	1.03 (0.95, 1.12)	0.42	1.05 (0.96, 1.15)	0.28
Alcohol	0.70 (0.37, 1.32)	0.27	0.52 (0.25, 1.10)	0.09
Coffee	0.75 (0.40, 1.39)	0.36	0.91 (0.44, 1.85)	0.79
Tea	1.32 (0.71, 2.46)	0.07	1.46 (0.72, 2.98)	0.29
Exercise	1.10 (0.60, 2.04)	0.75	1.40 (0.69, 2.81)	0.35
PPI duration (days)	1.01 (1.00, 1.02)	0.04	1.01 (1.00, 1.03)	0.01
RDQ_t0_	1.03 (0.99, 1.06)	0.18	1.01 (0.97, 1.05)	0.65
PSQI	1.07 (0.98, 1.17)	0.12	1.11 (1.00, 1.24)	0.04

Dependent variable: y = 1 for ∆TDQ < 0 and y = 0 for ∆TDQ ≥ 0. CI, confidence interval; PPI, proton-pump inhibitors; PSQI, Pittsburgh sleep quality index; RDQ, reflux disease questionnaire; t_0_, baseline. ^a^ Adjusted for mucosal break, gender, age, level of education, marriage, body mass index, alcohol, coffee, tea, exercise, PPI duration, RDQ_t0_ score, and PSQI score.

## Data Availability

The data presented in this study are available on a request from the corresponding author for researchers who meet the criteria for access to confidential data.
